# Supplementary material for: Morphological bases of phytoplankton energy management and physiological responses unveiled by 3D subcellular imaging
Source: Nat Commun. 2021 Feb 16;12:1049. doi: 10.1038/s41467-021-21314-0 (PMC7886885; doi:10.1038/s41467-021-21314-0)
Supplement: Supplementary file 1 — Supplementary Information [file 41467_2021_21314_MOESM1_ESM.pdf]

Supplementary Information

Morphological bases of phytoplankton energy management and physiological responses unveiled by 3D subcellular imaging

Clarisse Uwizeye[1], Johan Decelle[1]*, Pierre-Henri Jouneau[2], Serena Flori[1,3] Benoit Gallet[4], Jean-Baptiste Keck[5], Davide dal Bo[1], Christine Moriscot[4,6], Claire Seydoux[1], Fabien Chevalier[1], Nicole L. Schieber[7], Rachel Templin[7], Guillaume Allorent[1], Florence Courtois[1], Gilles Curien[1], Yannick Schwab[7,8], Guy Schoehn[4], Samuel C. Zeeman[9], Denis Falconet[1]*, Giovanni Finazzi[1]*.

[1]Univ. Grenoble Alpes, CNRS, CEA, INRAe, IRIG-LPCV, 38000 Grenoble, FRANCE.

[2]Univ. Grenoble Alpes, CEA, IRIG-MEM, 38000 Grenoble, FRANCE.

[3]The Marine Biological Association, The Laboratory, Citadel Hill Plymouth, Devon, PL1 2PB, UK

[4]Univ. Grenoble Alpes, CNRS, CEA, IRIG-IBS, 38000 Grenoble, FRANCE.

[5]Univ. Grenoble Alpes, Laboratoire Jean Kuntzmann, 38000 Grenoble, FRANCE.

[6]Univ. Grenoble Alpes, CNRS, CEA, EMBL, Integrated Structural Biology Grenoble (ISBG) 38000, Grenoble, FRANCE.

[7]Cell Biology and Biophysics Unit, European Molecular Biology Laboratory, 69117 Heidelberg, GERMANY.

[8]Electron Microscopy Core Facility, European Molecular Biology Laboratory, 69117 Heidelberg, GERMANY.

[9]Institute of Molecular Plant Biology, Department of Biology, ETH Zurich, 8092 Zurich, SWITZERLAND.

Supplementary Figures

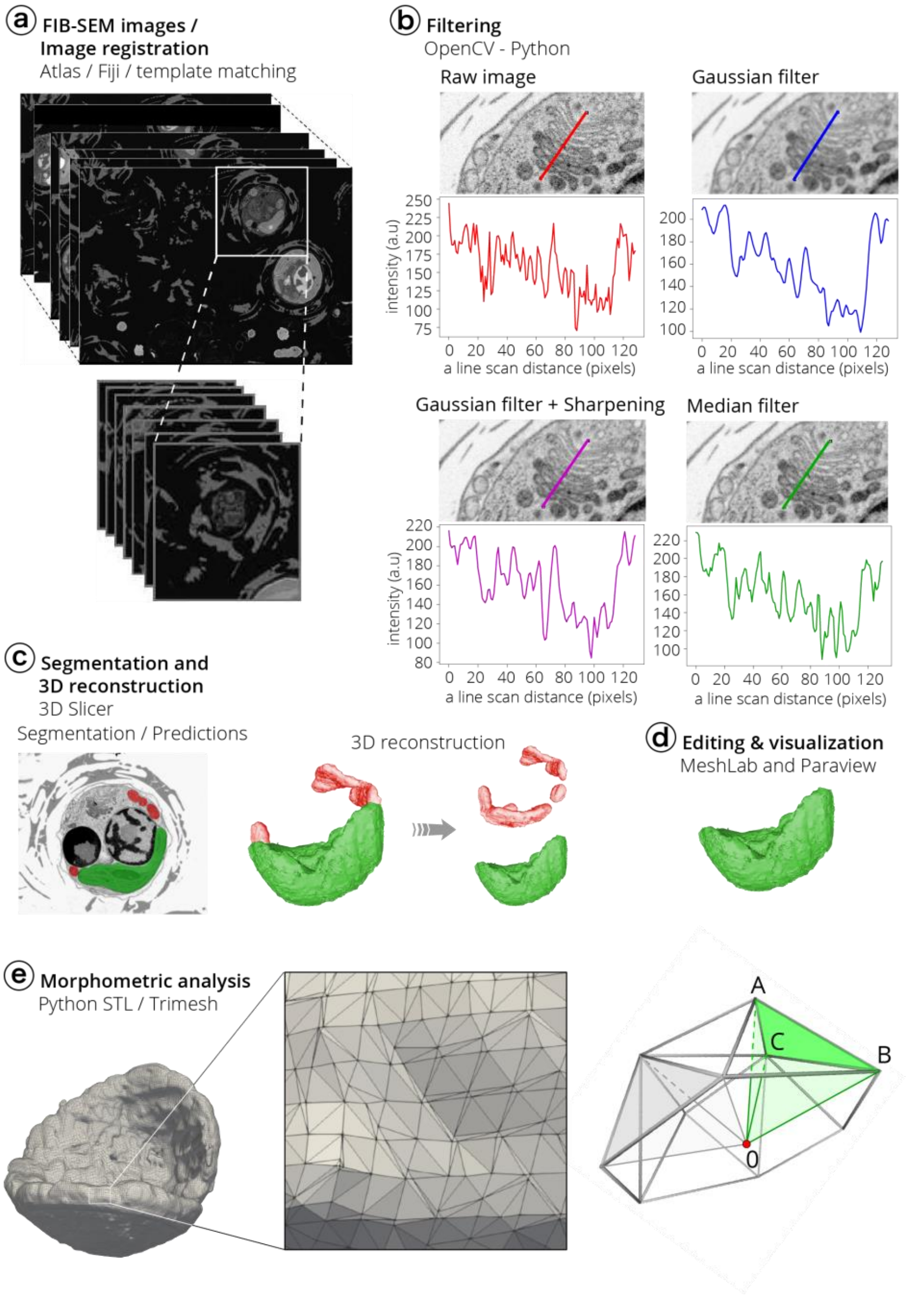

Supplementary Fig. 1: Flowchart of image processing from data acquisition to 3D reconstruction and morphometric analysis.

a: The pipeline includes data acquisition with FIB-SEM and registration with Fiji. **b:** Single cells are selected from the whole FIB-SEM stacks, images are registered before inverting their contrast. Stacks are filtered in Python using the PyOpenCV module. Linear (Gaussian) filter followed by edge enhancement (sharpening) or non-linear filter (median filter) are suitable in different species based on their cellular features (see text). A scan line of the Golgi apparatus drawn with Fiji in *Emiliania huxleyi* shows the impact of different filters on the profile plot of the flattened membrane-enclosed disks (the cisternae). Red: original image. Blue: the Gaussian filter smooths the edges and some membranes disappear. Purple: sharpening after application of the Gaussian filter allows recovery of some image details after smoothing edges. Green: The median filter is less sensitive to edges. Sections are representatives micrographs of an experiment repeated three times with similar results**. c:** Image processing was done with 3D Slicer for segmentation and **d:** MeshLab and Paraview, for editing and visualisation, respectively. **e:** The STL and Trimesh python packages were used to quantify volumes, surfaces and distances. From a watertight mesh, surface is obtained by summing the surface of each individual triangle present in the mesh. Volume is computed according to ref[1].

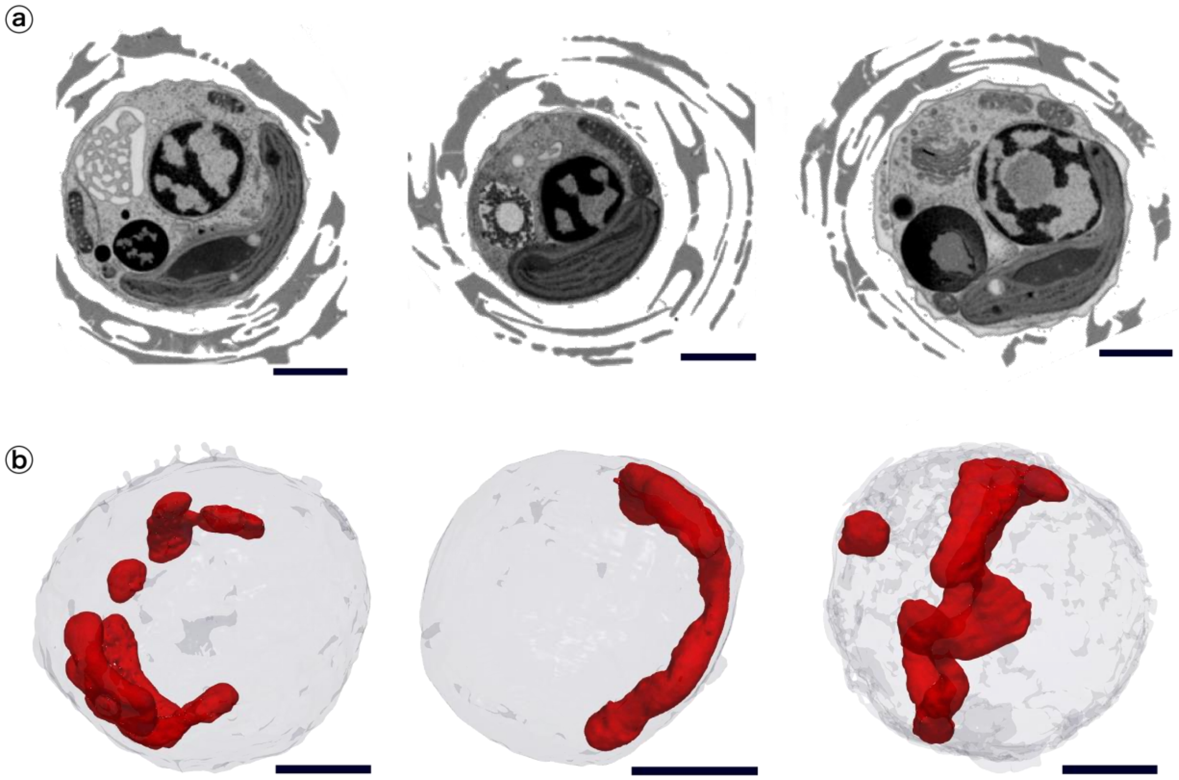

Supplementary Fig. 2: The different morphologies of mitochondria in three *Emiliania* (Prymnesiophyceae) cells.

a: Sections through cellular 3D volumes. Scale bar: 1 µm. **b**: Segmentations highlight the different 3D topology of the mitochondria (red) in the cell (light grey), consistent with the dynamic character of these organelles. Scale bar: 1 µm. Representatives micrographs and tomograms of an experiment repeated three times with similar results.

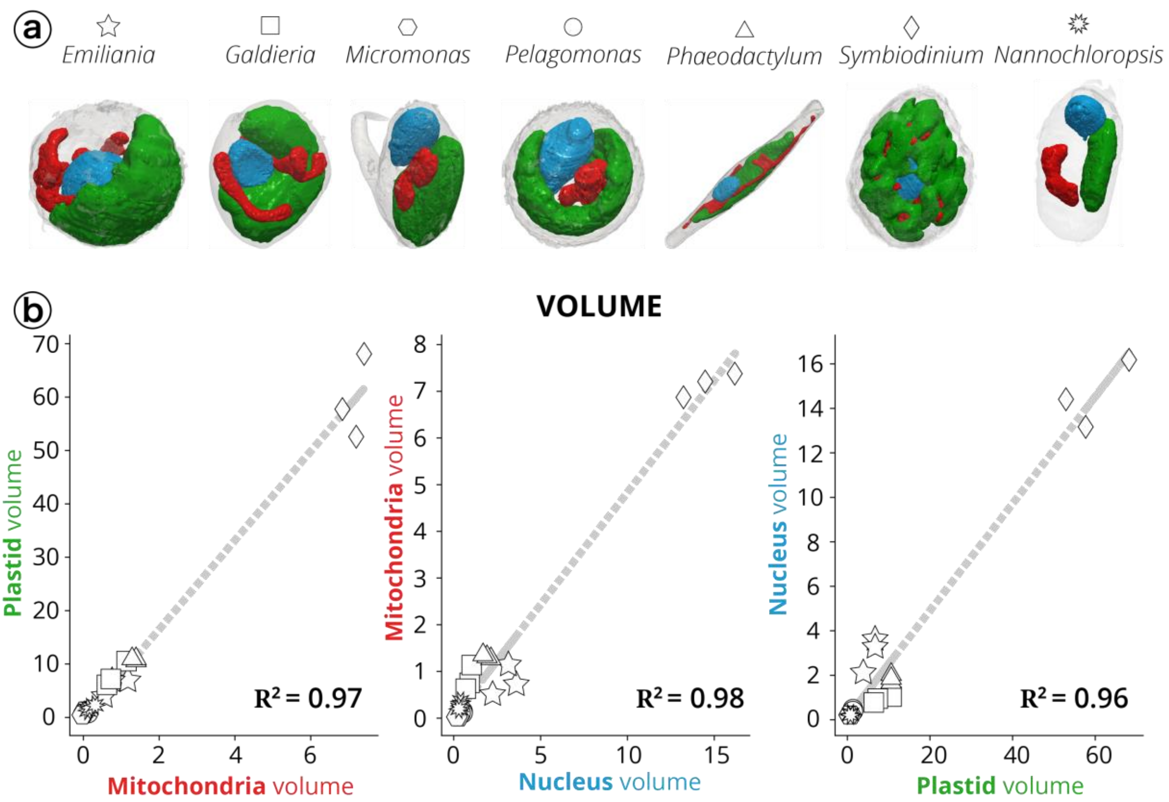

Supplementary Fig. 3: Volumes and surfaces relationship in different subcellular compartments, as derived from quantitative analysis of microalgal 3D models.

Three cells are considered for every species. **a:** stars: *Emiliania;* squares: *Galdieria*; hexagons: *Micromonas*; circles: *Pelagomonas*; triangles: *Phaeodactylum*; diamonds: *Symbiodinium; suns: Nannochloropsis.* **b:** Because of the much larger size of *Symbiodinium* cells, all the other taxa are compacted in a bottom left cluster in the plot. The presence of these two clusters prevents the observation of correlation between the other cells (Fig. 3).

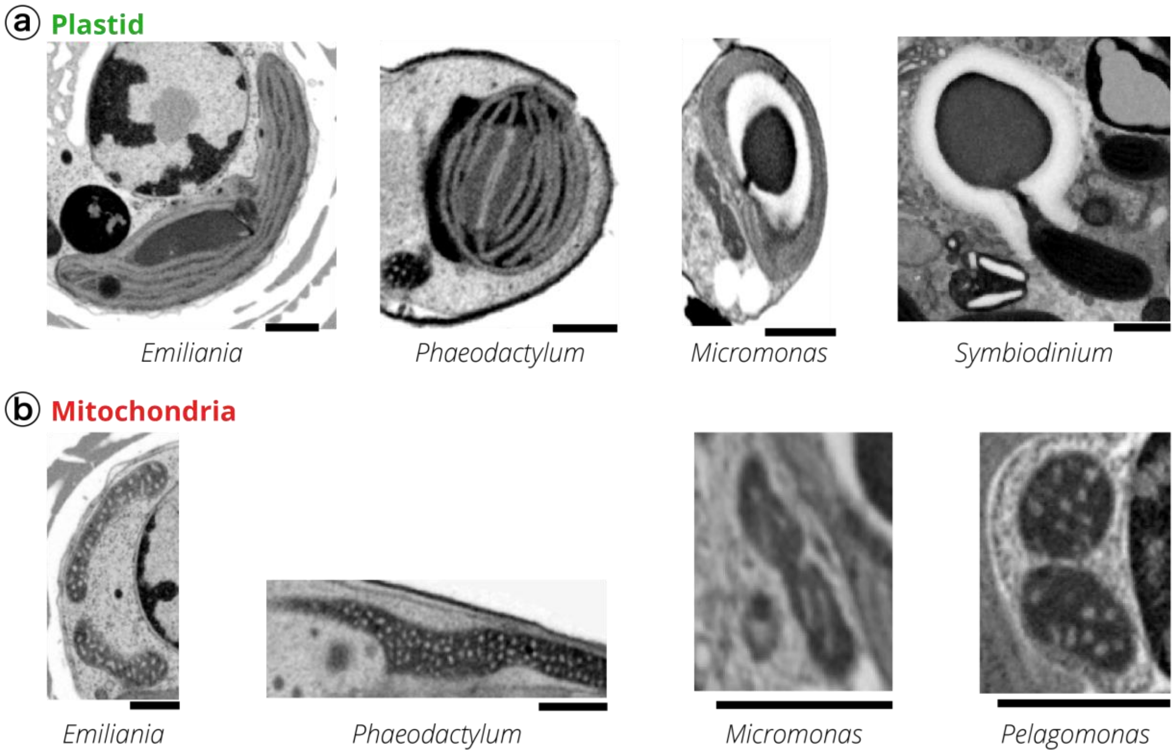

Supplementary Fig. 4: Subcellular features of different phytoplankton taxa*.*
Sections through cellular 3D volumes, segmented from FIB-SEM images of **a:** plastids, scale bar: 500 nm, **b:** mitochondria, scale bar: 500 nm. Representatives micrographs of an experiment repeated three times with similar results.

Supplementary Table 1.

Information on the phytoplankton taxa analyzed in this study (class, species and length size or diameter).

**Class**	**Species**	**Cell size**	**Fv/Fm** (this study)	**Fv/Fm** (literature)
Bacillariophyceae	*Phaeodactylum Pt1 8.6*	13-15 µm of length	0.61 ± 0.03	0.66 ± 0.03[3]
Pelagophyceae	*Pelagomonas RCC 100*	≤ 3 µm in diameter	0.48 ± 0.09	0.48 ± 0.01[4]
Dinophyceae	*Symbiodinium* (clade A) *RCC 4014*	7-8 µm in diameter	0.57 ± 0.04	0.55 ± 0.03[5]
Prymnesiophyceae	*Emiliania RCC 909*	3-8 µm in diameter	0.54 ± 0.05	0.57 ± 0.01[6]
Mammiellophyceae	*Micromonas RCC 827*	< 2 µm in diameter	0.67 ± 0.06	0.65 ± 0.00[7]
Cyanidiophyceae	*Galdieria SAG21.92*	3-9 µm	0.50 ± 0.01	0.50 ± 0.01[8]
Eustigmatophyceae	*Nannochloropsis CCMP 526*	2-3 µm	0.67 ± 0.15	0.60 ± 0.04[9]

The parameter Fv/Fm (maximum yield of photosystem II photochemistry[2]) was used to compare the photosynthetic capacity of the tested strain before cryofixation with earlier data in the literature, as a proxy for their physiological state.

Supplementary Table 2.

Comparison of standard 3D mesh formats in MeshLab in the case of *Symbiodinium* cells.

**3D output formats from 3D Slicer**	**.stl format***		**.obj format***		**.ply format***		**.vtk format***	
	**Before remesh**	**After remesh**	**Before remesh**	**After remesh**	**Before remesh**	**After remesh**	**Before remesh**	**After remesh**
**3D model file size**	583.4 Mb	100 Mb	1 Gb	178.9 Mb	223.6 Mb	36.6 Mb	256.6 Mb	-
**Model polygons**	7816626	2000000	7816626	2000000	7816626	2000000	-	-
**Volume information**	201.5 μm^3	201.5 μm^3	Watertight problem**	-	Watertight problem**	-	-	-

MeshLab is able to read almost all mesh formats (.obj; .stl; .ply) but not the .vtk format generated by 3Dslicer. While both the .obj and .ply files are smaller in size (and therefore easier to handle for visualization and animation), MeshLab encounter watertight problems with these formats (**: mesh surface is not closed). Therefore, quantitative analyses of volumes and surfaces were not possible unless using the .stl file format. After remeshing, Meshlab successfully reduced the number of polygons contained in the various objects, thereby generating smaller files that were easier to handle because of their reduced memory footprint. In addition, we checked that this mesh simplification procedure does not alter the volumetric information.

Supplementary Table 3.
The volume and surface of 3D models of plastids in *Emiliania.*

Geometrical analysis	3D Slicer (nrrd file)	Python based computing (STL file)	MeshLab (STL file)
Vol. model test (μm^3)	1.166	1.173	1.182
Surf. model test (μm^2)	Not provided	20.992	20.89

The volume and surface were computed using 3D Slicer software and compared with those obtained using the STL python package and MeshLab software.

References

1. Zhang, C., Chen, T. Efficient feature extraction for 2D/3D objects in mesh representation. In: *Proceedings 2001 International Conference on Image Processing (Cat. No. 01CH37205)*). IEEE (2001).

2. Butler, W. L. Energy distribution in the photochemical apparatus of photosynthesis. *Annual Review of Plant Physiology* **29**, 345-378 (1978).

3. Taddei, L*., et al.* Multisignal control of expression of the LHCX protein family in the marine diatom *Phaeodactylum tricornutum*. *Journal of experimental botany* **67**, 3939-3951 (2016).

4. Dimier, C., Brunet, C., Geider, R., Raven, J. Growth and photoregulation dynamics of the picoeukaryote *Pelagomonas calceolata* in fluctuating light. *Limnology and Oceanography* **54**, 823-836 (2009).

5. Tolleter, D*., et al.* Coral bleaching independent of photosynthetic activity. *Current Biology* **23**, 1782-1786 (2013).

6. Loebl, M., Cockshutt, A. M., Campbell, D. A., Finkel, V, Z. Physiological basis for high resistance to photoinhibition under nitrogen depletion in *Emiliania huxleyi*. *Limnology and Oceanography* **55**, 2150-2160 (2010).

7. Maat, D. S., de Blok, R., Brussaard, C. P. Combined phosphorus limitation and light stress prevent viral proliferation in the phytoplankton species *Phaeocystis globosa*, but not in *Micromonas pusilla*. *Frontiers in Marine Science* **3**, 160 (2016).

8. Oesterhelt, C., Schmälzlin, E., Schmitt, J. M., Lokstein, H. Regulation of photosynthesis in the unicellular acidophilic red alga *Galdieria sulphuraria*. *The Plant Journal* **51**, 500-511 (2007).

9. Simionato, D*., et al.* The response of *Nannochloropsis gaditana* to nitrogen starvation includes de novo biosynthesis of triacylglycerols, a decrease of chloroplast galactolipids, and reorganization of the photosynthetic apparatus. *Eukaryotic cell* **12**, 665-676 (2013).
