# Supplementary material for: Morphological bases of phytoplankton energy management and physiological responses unveiled by 3D subcellular imaging
Source: Nat Commun. 2021 Feb 16;12:1049. doi: 10.1038/s41467-021-21314-0 (PMC7886885; doi:10.1038/s41467-021-21314-0)
Supplement: Supplementary file 3 — Descriptions of Additional Supplementary Files [file 41467_2021_21314_MOESM3_ESM.pdf]

Descriptions of Additional Supplementary Files

Supplementary Data 1

Description: Quantitative analysis of the morphological features of different phytoplankton cells.

Supplementary data 2

Description: Python script for metrics computation (volumes, areas).

Supplementary data 3

Description: Python script to compute the minimal distance between two meshes (i.e. organelles proximity).

Supplementary Movie 1

Description: Focus Ion Beam Scanning Electron Microscopy (FIB-SEM) based 3D reconstruction of a whole cell of Micromonas RCC 827.

Supplementary Movie 2

Description: Focus Ion Beam Scanning Electron Microscopy (FIB-SEM) based 3D reconstruction of a whole cell of Pelagomonas RCC 100.

Supplementary Movie 3

Description: Focus Ion Beam Scanning Electron Microscopy (FIB-SEM) based 3D reconstruction of a whole cell of Nannochloropsis CCMP 526 (phototrophic conditions).

Supplementary Movie 4

Description: Focus Ion Beam Scanning Electron Microscopy (FIB-SEM) based 3D reconstruction of a whole cell of Galdieria SAG 21.92.

Supplementary Movie 5

Description: Focus Ion Beam Scanning Electron Microscopy (FIB-SEM) based 3D reconstruction of a whole cell of Emiliania RCC 909.

Supplementary Movie 6

Description: Focus Ion Beam Scanning Electron Microscopy (FIB-SEM) based 3D reconstruction of a whole cell of Phaeodactylum Pt1 8.6 (low light acclimated).

Supplementary Movie 7

Description: Focus Ion Beam Scanning Electron Microscopy (FIB-SEM) based 3D reconstruction of a whole cell of Symbiodinium RCC 4014 clade A.

Supplementary Movie 8

Description: Focus Ion Beam Scanning Electron Microscopy (FIB-SEM) based 3D reconstruction of a whole cell of Phaeodactylum Pt1 8.6 (high light acclimated).

Supplementary Movie 9

Description: Focus Ion Beam Scanning Electron Microscopy (FIB-SEM) based 3D reconstruction of a whole cell of Nannochloropsis CCMP 526 (mixotrophic conditions).

Supplementary Movie 10

Description: 3D representation of Focus Ion Beam Scanning Electron Microscopy (FIBSEM) based 3D reconstruction of a whole cell of Emiliania RCC 909. The video allows identifying inside the cell most of the known elements involved in the process of biomineralization known as coccolithogenesis. Grey: coccosphere (exoskeleton); blue: nucleus (dark blue: heavy-stained chromatin; light blue: low-stained chromatin); violet: nucleolus; green: plastid; red: mitochondria; purple: Golgi apparatus and other vesicles; yellow: storage compartments
