# Supplementary material for: Morphological bases of phytoplankton energy management and physiological responses unveiled by 3D subcellular imaging
Source: Nat Commun. 2021 Feb 16;12:1049. doi: 10.1038/s41467-021-21314-0 (PMC7886885; doi:10.1038/s41467-021-21314-0)
Supplement: Supplementary file 5 — Supplementary Data 2 [file 41467_2021_21314_MOESM5_ESM.docx]

**Supplementary dataset 2.** **Python script for metrics computation (volumes, areas)**

#-----------------------------------------------------------------------#

**import** numpy **as** np

**from** stl **import** mesh

mesh_file **=** 'a.stl'

mesh **=** mesh**.**Mesh**.**from_file**(**mesh_file**)**

**def** compute_surface**(**mesh**):**

surface **=** 0.0

**for** triangle **in** mesh**.**vectors**:**

X0**,**X1**,**X2 **=** triangle

u**,** v **=** X1**-**X0**,** X2**-**X0

w **=** np**.**cross**(**u**,** v**)**

surface **+=** np**.**sqrt**(**np**.**dot**(**w**,** w**))** **/** 2.0

**return** surface

**def** compute_volume**(**mesh**):**

volume **=** 0.0

**for** triangle **in** mesh**.**vectors**:**

X0**,**X1**,**X2 **=** triangle

volume **+=** np**.**dot**(**X0**,** np**.**cross**(**X1**,** X2**))** **/** 6.0

**return** volume

**def** compute_surface_fast**(**mesh**):**

Vt **=** mesh**.**vectors

U **=** Vt**[:,**1**]-**Vt**[:,**0**]**

V **=** Vt**[:,**2**]-**Vt**[:,**0**]**

W **=** np**.**cross**(**U**,**V**)**

S **=** np**.sum(**np**.**sqrt**(**np**.sum(**W*****W**,** axis**=**1**)))** **/** 2.0

**return** S

**def** compute_volume_fast**(**mesh**):**

Vt **=** mesh**.**vectors

V **=** np**.sum(**Vt**[:,**0**]***np**.**cross**(**Vt**[:,**1**],** Vt**[:,**2**]))** **/** 6.0

**return** V

shape **=** mesh**.**vectors**.**shape

pts **=** mesh**.**vectors**.**reshape**(**shape**[**0**]***shape**[**1**],** shape**[**2**])**

pmin **=** np**.min(**pts**,** axis**=**0**)**

pmax **=** np**.max(**pts**,** axis**=**0**)**

L **=** pmax **-** pmin

S **=** compute_surface_fast**(**mesh**)**

V **=** compute_volume_fast**(**mesh**)**

**print** '''

Mesh '{}' statistics:

pmin: {}

pmax: {}

L: {}

S: {}

V: {}

S/V: {}

'''**.format(**mesh_file**,** pmin**,** pmax**,** L**,** S**,** V**,** S**/**V**)**
