# Supplementary material for: Morphological bases of phytoplankton energy management and physiological responses unveiled by 3D subcellular imaging
Source: Nat Commun. 2021 Feb 16;12:1049. doi: 10.1038/s41467-021-21314-0 (PMC7886885; doi:10.1038/s41467-021-21314-0)
Supplement: Supplementary file 6 — Supplementary Data 3 [file 41467_2021_21314_MOESM6_ESM.docx]

**Supplementary dataset 3:**  **Python script to compute the minimal distance between two meshes (i.e. organelles contacts areas)**

#-------------------------------------------------------#

#!/usr/bin/env python3

**import** trimesh**,** argparse**,** os**,** sys**,** random**,** locale

**from** trimesh**.**proximity **import** closest_point **as** distance

**from** trimesh**.**visual **import** create_visual**,** linear_color_map

**from** matplotlib **import** pyplot **as** plt

**import** matplotlib**.**colors **as** mcolors

**from** bashplotlib**.**histogram **import** plot_hist

**from** texttable **import** Texttable

**import** numpy **as** np

np**.**set_printoptions**(**edgeitems**=**30**,** linewidth**=**100000**,**

formatter**=dict(float=lambda** x**:** "%.3g" **%** x**))**

locale**.**setlocale**(**locale**.**LC_ALL**,** 'en_GB.utf8'**)**

**def** color**(***args**,** ******kwargs**):**

color **=** np**.**array**([***args**],** dtype**=**np**.**uint8**)**

**if** 'n' **in** kwargs**:**

color **=** np**.**tile**(**color**,** **(**kwargs**[**'n'**],** 1**))**

**return** color

**def** flt**(**value**,** args**):**

**return** locale**.format(**'%.{p}f'**.format(**p**=**args**.**prec**),** value**,** **True)**

**def** minsurf**(**args**):**

prec **=** args**.**prec

**def** vprint**(***_args**,** ******_kwargs**):**

**if** args**.**verbose**:**

**print(***_args**,** ******_kwargs**)**

vprint**(**"Loading mesh0 '{}'..."**.format(**args**.**mesh0**))**

mesh0 **=** trimesh**.**load**(**args**.**mesh0**)**

vprint**(**"Loading mesh1 '{}'. "**.format(**args**.**mesh1**))**

mesh1 **=** trimesh**.**load**(**args**.**mesh1**)**

# apply custom user space scaling

mesh0**.**vertices ***=** args**.**space_scaling

mesh1**.**vertices ***=** args**.**space_scaling

vprint**(**"Surface statistics:"**)**

vprint**(**" mesh0: {} nm^2"**.format(**flt**(**mesh0**.**area**,**args**)))**

vprint**(**" mesh1: {} nm^2"**.format(**flt**(**mesh1**.**area**,**args**)))**

vprint**()**

vprint**(**"Computing distances..."**)**

**(**pts**,** dist**,** tid**)** **=** distance**(**mesh0**,** mesh1**.**vertices**)**

**if** args**.**verbose**:**

plot_hist**(**dist**,** pch**=**'|'**,** bincount**=**20**,** xlab**=True)**

vprint**(**'''

Global statistics:

min: {} nm

max: {} nm

mean: {} nm

'''**.format(**flt**(**dist**.min(),**args**),** flt**(**dist**.max(),**args**),** flt**(**dist**.**mean**(),**args**)))**

**if** **(**args**.**max_distance **is** **None):**

**if** args**.**compute_distribution**:**

args**.**max_distance **=** np**.**inf

**else:**

msg**=**'No max distance has been given, exiting.'

vprint**(**msg**)**

sys**.exit(**0**)**

vprint**(**"Extracting vertices closest than {} nm..."**.format(**args**.**max_distance**))**

node_ids **=** np**.**where**(**dist**<=**args**.**max_distance**)[**0**]**

**if** **(**node_ids**.**size **==** 0**):**

msg**=**'No vertex meeting this criteria has been found, aborting.'

vprint**(**msg**)**

sys**.exit(**0**)**

kwds **=** **dict(**args**=**args**,** mesh0**=**mesh0**,** mesh1**=**mesh1**,** pts**=**pts**,**

dist**=**dist**,** tid**=**tid**,** node_ids**=**node_ids**,** vprint**=**vprint**)**

**if** args**.**compute_distribution**:**

compute_distribution**(****kwds**)**

**else:**

extract_close_faces**(****kwds**)**

**def** extract_faces**(**faces**,** node_ids**,** all_faces**):**

**if** all_faces**:**

is_close **=** **lambda** face**:** **any((**nid **in** node_ids**)** **for** nid **in** face**)**

**else:**

is_close **=** **lambda** face**:** **all((**nid **in** node_ids**)** **for** nid **in** face**)**

**return** **tuple(filter(**is_close**,** faces**))**

**def** compute_distribution**(**args**,** mesh0**,** mesh1**,** pts**,** dist**,** tid**,** node_ids**,** vprint**):**

vprint**(**'Computing distribution of surface bellow {:.{p}f} nm^2...'**.format(**args**.**max_distance**,** p**=**args**.**prec**))**

distance **=** dist**[**node_ids**]**

mind**,** maxd **=** np**.**floor**(**distance**.min()),** np**.**ceil**(**distance**.max())**

S **=** mesh1**.**area

D **=** np**.**linspace**(**mind**,** maxd**,** args**.**npts**)**

A **=** np**.**full_like**(**D**,** fill_value**=**np**.**nan**)**

**for** **(**i**,**d**)** **in** **enumerate(**D**):**

node_ids **=** **frozenset(**np**.**where**(**dist**<=**d**)[**0**])**

faces **=** extract_faces**(**mesh1**.**faces**,** node_ids**,** args**.**all_faces**)**

**if** faces**:**

A**[**i**]** **=** trimesh**.**Trimesh**(**vertices**=**mesh1**.**vertices**,** faces**=**faces**).**area

**else:**

A**[**i**]** **=** 0.0

P **=** 100***(**A**/**S**)**

vprint**(**'Surface distribution:'**)**

vprint**(**' {}'**.format(**D**))**

vprint**(**' {}'**.format(**P**))**

**if** args**.**do_plot**:**

plt**.**plot**(**D**,**P**)**

plt**.**scatter**(**D**,**P**)**

plt**.**xlabel**(**'distance (nm)'**)**

plt**.**ylabel**(**'percentage of total area ({:.{p}f} nm^2)'**.format(**S**,** p**=**args**.**prec**))**

plt**.**xlim**(**mind**,**maxd**)**

plt**.**ylim**(**0**,**P**.max())**

plt**.**show**()**

**def** extract_close_faces**(**args**,** mesh0**,** mesh1**,** pts**,** dist**,** tid**,** node_ids**,** vprint**):**

prec **=** args**.**prec

snode_ids **=** **frozenset(**node_ids**.**tolist**())**

**if** args**.**all_faces**:**

vprint**(**"Extracting faces containing at least one close node..."**)**

is_close **=** **lambda** face**:** **any((**nid **in** snode_ids**)** **for** nid **in** face**)**

**else:**

vprint**(**"Extracting faces containing only close nodes..."**)**

is_close **=** **lambda** face**:** **all((**nid **in** snode_ids**)** **for** nid **in** face**)**

faces **=** np**.**asarray**(tuple(filter(**is_close**,** mesh1**.**faces**)))**

vprint**(**"Extracting vertices..."**)**

mesh_node_ids **=** **tuple(sorted(**np**.**unique**(**faces**.**ravel**())))**

mesh_node_pos **=** mesh1**.**vertices**[**np**.**asarray**(**mesh_node_ids**)]**

**(**mesh_pts**,** mesh_dist**,** mesh_tid**)** **=** distance**(**mesh0**,** mesh_node_pos**)**

vprint**(**"Reindexing vertices..."**)**

reindex **=** **lambda** face**:** **tuple(**mesh_node_ids**.**index**(**i**)** **for** i **in** face**)**

mesh_faces **=** **tuple(map(**reindex**,** faces**))**

mesh **=** trimesh**.**Trimesh**(**vertices**=**mesh_node_pos**,** faces**=**mesh_faces**)**

# print statistics about extracted data

vprint**(**''**)**

vprint**(**'Successfully extracted {} faces defined by {} nodes:'**.format(len(**mesh**.**faces**),** **len(**mesh**.**vertices**)))**

vprint**(**' min dist: {} nm'**.format(**flt**(**mesh_dist**.min(),**args**)))**

vprint**(**' max dist: {} nm'**.format(**flt**(**mesh_dist**.max(),**args**)))**

vprint**(**' mean dist: {} nm'**.format(**flt**(**mesh_dist**.**mean**(),**args**)))**

vprint**(**''**)**

msg1**=**"Mesh '{}' surface"**.format(**args**.**mesh1**.**split**(**'/'**)[-**1**])**

msg2**=**"area <= max_dist"

msg3**=**"percentage"

msg2**=**"{}{}"**.format(**' '***(len(**msg1**)-len(**msg2**)),** msg2**)**

msg3**=**"{}{}"**.format(**' '***(len(**msg1**)-len(**msg3**)),** msg3**)**

vprint**(**'{}: {} nm^2'**.format(**msg1**,** flt**(**mesh1**.**area**,**args**)))**

vprint**(**'{}: {} nm^2'**.format(**msg2**,** flt**(**mesh**.**area**,**args**)))**

vprint**(**'{}: {:.{p}f}%'**.format(**msg3**,** **(**100.0*****mesh**.**area**)/**mesh1**.**area**,** p**=**prec**))**

# we stop here if 3D plot was not asked explicitely by user

**if** **not** args**.**do_plot**:**

**return**

mesh**.**vertices**[...]** ***=** args**.**plot_scaling

mesh0**.**vertices**[...]** ***=** args**.**plot_scaling

mesh1**.**vertices**[...]** ***=** args**.**plot_scaling

mesh_pts**[...]** ***=** args**.**plot_scaling

# determine mesh0 base color (color applied to faces or vertices that are too far)

**if** **(**args**.**mesh_color **is** **None):**

mesh_color**(**0**,**0**,**128**,)**

**else:**

mesh_color **=** **tuple(**255*****_ **for** _ **in** mcolors**.**to_rgb**(**args**.**mesh_color**))**

mesh_color **+=** **(**args**.**mesh_alpha*****255**,)** # alpha channel

# determine mesh1 base color (color applied to faces or vertices that are too far)

**if** **(**args**.**base_color **is** **None):**

base_color **=** **(**128**,**128**,**128**,)**

**else:**

base_color **=** **tuple(**255*****_ **for** _ **in** mcolors**.**to_rgb**(**args**.**base_color**))**

base_color **+=** **(**args**.**base_alpha*****255**,)** # alpha channel

# use full dist if displaying the whole mesh, else use reduced dist

**if** **(**args**.**display_mode **==** 'minimal'**):**

display_mesh **=** mesh

vertex_dist **=** mesh_dist

**else:**

display_mesh **=** mesh1

vertex_dist **=** dist

face_dist **=** vertex_dist**[**display_mesh**.**faces**].max(**axis**=**1**)**

dist **=** vertex_dist **if** args**.**use_vertex_colors **else** face_dist

# here there are two modes: linear color interpolation from distance or a

# subdivision based mode.

**if** **(**args**.**subdivisions **is** **None):**

values **=** **(**dist**-**dist**.min())/(**dist**.max()-**dist**.min())**

mdval **=** **(**args**.**max_distance **-** dist**.min())/(**dist**.max()-**dist**.min())**

values **/=** mdval

values **=** np**.**minimum**(**values**,** 1.0**)**

**if** **(**args**.**custom_colors **is** **not** **None):**

**assert** **len(**args**.**custom_colors**)** **==** 2

colors **=** **tuple(map(lambda** c**:** **tuple(int(**ci*****255**)**

**for** ci **in** mcolors**.**to_rgba**(**c**)),** args**.**custom_colors**))**

**else:**

colors **=** **([**255**,**0**,**0**,**255**],[**0**,**255**,**0**,**255**])**

colors **=** linear_color_map**(**values**,** color**(***colors**))**

**else:**

subdivisions **=** args**.**subdivisions

**assert(**subdivisions**>=**1**)**

**if** **(**args**.**custom_colors **is** **not** **None):**

**assert** **len(**args**.**custom_colors**)** **>=** subdivisions

colors **=** args**.**custom_colors

**elif** subdivisions**<=**6**:**

colors **=** mcolors**.**BASE_COLORS

**elif** subdivisions**<=**10**:**

colors **=** mcolors**.**TABLEAU_COLORS

**else:**

colors **=** **list(**mcolors**.**CSS4_COLORS**)**

random**.**shuffle**(**colors**)**

subdivision_colors **=** **tuple(map(lambda** c**:** **tuple(int(**ci*****255**)**

**for** ci **in** mcolors**.**to_rgba**(**c**)),** colors**))**

d **=** **(**dist**-**dist**.min())/(**args**.**max_distance**-**dist**.min())**

reldist **=** np**.**linspace**(**0.0**,** 1.0**,** subdivisions**+**1**)**

colors **=** np**.**empty**(**shape**=(**d**.**size**,** 4**),** dtype**=**np**.**uint8**)**

colors**[...]** **=** np**.**asarray**(**base_color**)[None,:]**

vprint**()**

**for** i **in** **range(**subdivisions**):**

mask **=** np**.**logical_and**(**d**>=**reldist**[**i**],** d**<=**reldist**[**i**+**1**])**

colors**[**mask**]** **=** subdivision_colors**[**i**]**

# compute area

node_ids **=** **frozenset(**np**.**where**(**d**<=**reldist**[**i**+**1**])[**0**])**

faces **=** extract_faces**(**display_mesh**.**faces**,** node_ids**,** args**.**all_faces**)**

**if** faces**:**

A **=** trimesh**.**Trimesh**(**vertices**=**display_mesh**.**vertices**,** faces**=**faces**).**area

**else:**

A **=** 0.0

msg**=**'Area bellow {:6.{p}f}% of maxdist: A(d<={} nm) = {} nm^2'

vprint**(**msg**.format(**100*****reldist**[**i**+**1**],** flt**(**d**[**i**+**1**],**args**),** flt**(**A**,**args**),** p**=**prec**))**

**(**vertex_colors**,** face_colors**)** **=** **(**colors**,** **None)** **if** args**.**use_vertex_colors \

**else** **(None,** colors**)**

display_mesh**.**visual**.**vertex_colors **=** vertex_colors

display_mesh**.**visual**.**face_colors **=** face_colors

mesh0**.**visual **=** create_visual**(**mesh**=**mesh0**,** face_colors**=**mesh_color**)**

**if** args**.**display_mode **in** **(**'minimal'**,** 'default'**):**

trimesh_meshes **=** **[**display_mesh**]**

**if** args**.**use_vertex_colors**:**

scene **=** trimesh**.**Scene**(**trimesh_meshes**)**

scene**.**show**()**

**else:**

**from** vtkplotter **import** show**,** trimesh2vtk**,** addons

vtk_meshes **=** **[**trimesh2vtk**(**m**)** **for** m **in** trimesh_meshes**]**

show**(**vtk_meshes**,** bg**=**'white'**,** axes**=**0**)**

**elif** args**.**display_mode **==** 'all'**:**

colors **=** np**.**asarray**([**trimesh**.**visual**.**random_color**()** **for** _ **in** **range(**mesh_pts**.**size**)])**

cloud **=** trimesh**.**points**.**PointCloud**(**mesh_pts**,** colors**)**

display **=** **[**cloud**,** mesh0**,** display_mesh**]**

scene **=** trimesh**.**Scene**(**display**)**

scene**.**show**()**

**else:**

msg**=**'Unknown display mode \'{}\', please use minimal, default or all.'

**raise** **RuntimeError(**msg**.format(**args**.**display_mode**))**

**if** __name__ **==** '__main__'**:**

description**=**'Compute minimal distance between two STL meshes.'

epilog**=**''

parser **=** argparse**.**ArgumentParser**(**prog**=**'minsurf'**,**

description**=**description**,** epilog**=**epilog**)**

main_params **=** parser**.**add_argument_group**(**'Main parameters'**)**

main_params**.**add_argument**(type=str,** dest**=**'mesh0'**,** **help=**'Path to first input mesh.'**)**

main_params**.**add_argument**(type=str,** dest**=**'mesh1'**,** **help=**'Path to second input mesh.'**)**

main_params**.**add_argument**(**'-d'**,** '--max-distance'**,** **type=float,** default**=None,**

dest**=**'max_distance'**,** **help=**'Maximum distance of nodes that determine a close triangle.'**)**

main_params**.**add_argument**(**'-af'**,** '--all-faces'**,** action**=**'store_true'**,**

dest**=**'all_faces'**,** **help=**'Include faces that contain at least one close node instead of faces that contain only close nodes.'**)**

main_params**.**add_argument**(**'-cd'**,** '--compute-distribution'**,** action**=**'store_true'**,**

dest**=**'compute_distribution'**,** **help=**'Compute distribution of area with respect to distance.'**)**

main_params**.**add_argument**(**'-n'**,** '--npoints'**,** **type=int,** default**=**16**,**

dest**=**'npts'**,** **help=**'Number of points to compute distribution.'**)**

main_params**.**add_argument**(**'-ss'**,** '--space-scaling'**,** **help=**'Specify mesh unit (voxel size) in nm.'**,**

dest**=**'space_scaling'**,** **type=float,** default**=**1.0**)**

main_params**.**add_argument**(**'-ps'**,** '--plot-scaling'**,** **help=**'Specify scaling for plots.'**,**

dest**=**'plot_scaling'**,** **type=float,** default**=**1.0e-2**)**

main_params**.**add_argument**(**'-prec'**,** '--precision'**,** **help=**'Specify number of digits to be printed out.'**,**

dest**=**'prec'**,** **type=int,** default**=**2**)**

main_params**.**add_argument**(**'-nv'**,** '--no-verbose'**,** action**=**'store_false'**,**

dest**=**'verbose'**,** **help=**'Disable verbosity.'**)**

main_params**.**add_argument**(**'-plt'**,** '--plot'**,** action**=**'store_true'**,**

dest**=**'do_plot'**,** **help=**'Plot meshes and closest points.'**)**

main_params**.**add_argument**(**'-mc'**,** '--mesh-color'**,** **type=str,** default**=None,**

dest**=**'mesh_color'**,** **help=**'Other mesh color, defaults to gray. Use matplotlib colornames such as \'chartreuse\' or \'Melon\'.'**)**

main_params**.**add_argument**(**'-ma'**,** '--mesh-alpha'**,** **type=float,** default**=**1.0**,**

dest**=**'mesh_alpha'**,** **help=**'Other mesh alpha between 0.0 and 1.0, defaults to 100%%. '**)**

main_params**.**add_argument**(**'-bc'**,** '--base-color'**,** **type=str,** default**=None,**

dest**=**'base_color'**,** **help=**'Base mesh color, defaults to gray. Use matplotlib colornames such as \'chartreuse\' or \'Melon\'.'**)**

main_params**.**add_argument**(**'-ba'**,** '--base-alpha'**,** **type=float,** default**=**1.0**,**

dest**=**'base_alpha'**,** **help=**'Base mesh alpha between 0.0 and 1.0, defaults to 100%%. '**)**

main_params**.**add_argument**(**'-s'**,** '--subdivisions'**,** **type=int,** default**=None,**

dest**=**'subdivisions'**,** **help=**'Render only s colors for the distance map. When set to None, display a color gradient and ignore --custom-colors.'**)**

main_params**.**add_argument**(**'-cc'**,** '--custom-colors'**,** **type=str,** default**=None,**

dest**=**'custom_colors'**,** **help=**'Comma-separated list of custom distance dependent colors (see --subdivisions). Use color names from matplotlib (see --base-color).'**)**

main_params**.**add_argument**(**'-vc'**,** '--vertex-colors'**,** action**=**'store_true'**,**

dest**=**'use_vertex_colors'**,** **help=**'If set, use distance data to color vertices instead of whole faces. Face colors will be linearly interpolated from face nodes colors.'**)**

main_params**.**add_argument**(**'-dm'**,** '--display-mode'**,** **type=str,** default**=**'default'**,**

dest**=**'display_mode'**,** **help=(**'Choose what has to be displayed between minimal (displays only surface bellow max distance), '

'default (displays whole mesh including far faces) and all (displays both meshes and points).'**))**

args **=** parser**.**parse_args**()**

**if** **(**args**.**custom_colors **is** **not** **None):**

**if** **(**';' **in** args**.**custom_colors**)** **or** **(**' ' **in** args**.**custom_colors**):**

msg**=**'Multiple colors should be given as a comma separated list of colors, got {}.'

parser**.**error**(**msg**.format(**args**.**custom_colors**))**

args**.**custom_colors **=** args**.**custom_colors**.**split**(**','**)**

**for** stl **in** **(**args**.**mesh0**,** args**.**mesh1**):**

**if** **(len(**stl**)<**4**)** **or** **(**stl**[-**4**:]!=**'.stl'**):**

**raise** **ValueError(**'Input file \'{}\' does not match input file format \'*.stl\'.'**.format(**stl**))**

**if** **not** os**.**path**.**isfile**(**stl**):**

**raise** **ValueError(**'Input file \'{}\' does not exist.'**.format(**stl**))**

minsurf**(**args**)**
